# Host–parasite relationship in urban environments: A network analysis of haemoparasite infections in *Nasua nasua* Linnaeus (South American coati)

**DOI:** 10.1111/mve.12803

**Published:** 2025-03-24

**Authors:** Filipe Martins Santos, Nayara Yoshie Sano, Livia Perles, Marcos Rogério André

**Affiliations:** ^1^ Programa de Pós‐Graduação em Ciências Ambientais e Sustentabilidade Agropecuária Universidade Católica Dom Bosco (UCDB) Campo Grande Brazil; ^2^ Programa de Pós‐graduação em Microbiologia State University of Rio de Janeiro Rio de Janeiro Brazil; ^3^ Programa de Pós‐Graduação em Biotecnologia Universidade Católica Dom Bosco (UCDB) Campo Grande Brazil; ^4^ Vector‐Borne Bioagents Laboratory (VBBL), Departamento de Patologia, Reprodução e Saúde Única da Faculdade de Ciências Agrárias e Veterinárias Universidade Estadual Paulista (UNESP) Jaboticabal Brazil

**Keywords:** coati, network metrics, social behaviour, urban forest fragments

## Abstract

Parasite relationships are influenced by host size, behaviour, population density and location and can affect the ecological dynamics of their hosts. Urban environments provide new contexts for host–parasite interactions, often leading to changes in infection dynamics when compared with the natural environment. This study focuses on the relationship between five genera of haemoparasites (*Anaplasma* Theiler, *Ehrlichia* Moshkovski, *Hepatozoon* Miller, haemotropic *Mycoplasma* Nowak and *Neorickettsia* Philip) found in the South American coati *Nasua nasua* Linnaeus (Carnivora: Procyonidae), a carnivore highly adaptable to urban areas. Here, we used network analysis to verify the interaction between *N. nasua* and haemoparasites. We also used a General Linear Model to investigate the influence of biotic and abiotic variables and haemoparasite infections on the functional roles of *N. nasua* individuals, considering weight, age, sex and tick infestation (number of immature ticks collected). The network revealed low modularity, and none of the biotic variables, immature stages of ticks and location of sampling had any influence on the functional role of *N. nasua*. The most important haemoparasite in the network was haemotropic *Mycoplasma*, identified as a key non‐hub connector, probably spreading efficiently through frequent agonistic social interactions from *N. nasua*. These findings underscore the complex interplay between host behaviour, environmental factors and parasite ecology in urban environments, offering insights into managing urban wildlife diseases.

## Graphical abstract

- Urban environments alter host–parasite dynamics, as seen in *Nasua nasua*, where haemotropic *Mycoplasma* plays a key role in parasite spread through frequent social interactions.
- Network analysis revealed low modularity in interactions between *N. nasua* and tick‐borne haemoparasites, with functional roles unaffected by biotic factors, tick infestation or study area.
- Insights into host–parasite relationships in urban settings highlight the influence of host behaviour and environmental factors on disease ecology and wildlife management.

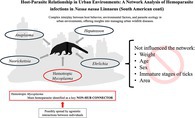

## Resumo

Relações entre parasitas podem ser influenciados pelo tamanho do hospedeiro, comportamento, densidade populacional e local, e pode afetar a dinâmica ecológica de seus hospedeiros. Ambientes urbanos provêm novos contextos para interação parasita‐hospedeiro, levando frequentemente a alterações na dinâmica de infecção quando comparado com o ambiente natural. O presente estudo focou na relação entre cinco gêneros de hemoparasitas (*Anaplasma* Theiler, *Ehrlichia* Moshkovski, *Hepatozoon* Miller, hemotropic *Mycoplasma* Nowak and *Neorickettsia* Philip) encontrados em quatis sul‐americanos *Nasua nasua* Linnaeus (Carnivora: Procyonidae), um carnívoro altamente adaptável a áreas urbanas. Aqui, nós utilizamos análise de rede para verificar a interação entre *N. nasua* e hemoparasitas. Além disso, nós usamos Modelos Lineares Generalizados para investigar a influência de variáveis bióticas e abióticas e infecção de hemoparasitas nos papéis funcionais para indivíduos de *N. nasua*, considerando peso, idade, sexo e infestação (número de carrapatos imaturos coletados). A rede mostrou baixa modularidade, e nenhuma das variáveis bióticas, estágios imaturos de carrapatos, e local de coleta tiveram influência no papel funcional de *N. nasua*. O hemoparasita mais importante na rede foi *Mycoplasma* hemotrófico, identificado como eixo não conector chave, provavelmente se espalhando eficientemente pela interação agonística de *N. nasua*. Esses achados mostram a interação complexa entre comportamento do hospedeiro, fatores ambientais e ecologia parasitária em ambientes urbanos, oferecendo novas percepções no manejo de doenças que acometem a fauna silvestre.

## INTRODUCTION

*Nasua nasua* Linnaeus (Carnivora: Procyonidae) South American coati, a medium‐sized carnivore member of the Procyonidae family, exhibits remarkable adaptability to urban and peri‐urban environments (Barreto et al., 2021, 2024; Gompper & Decker, 1998). These mammals take advantage of abundant food resources, such as domestic refuse, while encountering few natural predators (Barreto et al., 2024; Ferreira et al., 2013; Marques et al., 2008). Urban forest fragments serve as critical resting and foraging habitats due to their arboreal resting behaviour (Barreto et al., 2021, 2024). However, such environments also expose coatis to unique parasitic pressures, potentially altering the structure of host–parasite networks.

The diversity of parasite species detected in a host is influenced by multiple ecological factors, such as host size, location, population density and social behaviour (de Macedo et al., 2022; Oliveira‐Santos et al., 2021; Rademaker et al., 2009), as well as abiotic factors (Santos et al., 2022) in natural environments. Urbanisation has profoundly transformed these dynamics through habitat fragmentation, biodiversity loss and the simplification of ecosystems, creating novel ecological contexts that alter host–parasite interactions and infection dynamics (Soulsbury & White, 2015). Such changes often result in the introduction of non‐native species and the disruption of existing food chains, further influencing parasitic assemblages (Bradley & Altizer, 2007; McKinney, 2006). Parasite transmission in urban environments has direct and indirect mechanisms, including contact with infected hosts, exposure to contaminated environments or through vectors (Waleckx et al., 2015). These dynamics are particularly relevant for zoonotic parasites, which can spill over to domestic animals and humans, posing significant public health concerns. For instance, haemoparasites transmitted by arthropod vectors are of special interest due to their ability to infect a wide range of vertebrate hosts, including humans, and their potential to cause severe diseases (Roque & Jansen, 2014). Understanding how urbanisation affects these transmission pathways is crucial for addressing ecological and public health challenges.

Studying host–parasite interactions in urban South American coati populations offers valuable insights into how biodiversity and ecological dynamics are shaped by urbanisation. Network metrics provide powerful tools for examining these interactions, enabling an understanding of the structure and dynamics of ecological relationships in urban contexts (Mello et al., 2011, 2015, 2019; Santos & Sano, 2022a, 2022b). By applying these methods to assess host–parasite associations, it is possible to reveal complex interdependencies and their implications for both biodiversity and ecosystem health (Cardoso et al., 2021; Juárez‐Juárez et al., 2023). Here, we aimed to investigate the relationship between haemoparasites and individuals of South American coati sampled in urban forest fragments in the municipality of Campo Grande, state of Mato Grosso do Sul, central‐western Brazil. This study seeks to contribute to understanding how urbanisation influences parasitic dynamics and their potential zoonotic implications. Specifically, we hypothesise that urban environments, with their altered ecological interactions and increased habitat disturbance, may lead to a shift in the prevalence and diversity of parasites in South American coati, potentially increasing the risk of zoonotic transmission to other wildlife and humans. This work intends to provide insights into these dynamics and offer a deeper understanding of how urbanisation shapes parasite–host interactions in fragmented landscapes.

## MATERIALS AND METHODS

### Study area

**FIGURE 1:**
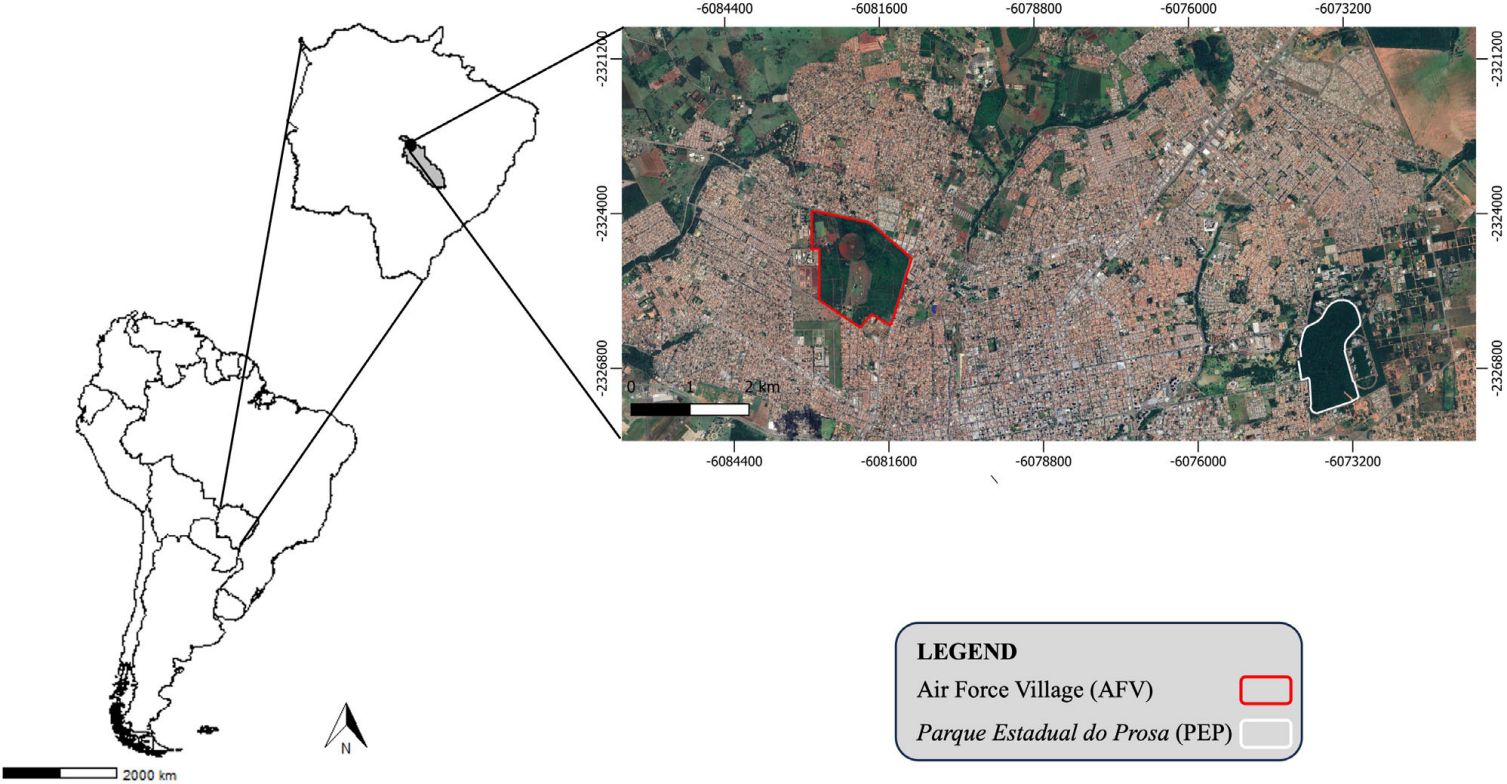
Map of South America showing the state of Mato Grosso do Sul, Brazil, with Campo Grande municipality highlighted in grey. In the panel on the right are the both studied areas: the *Parque Estadual do Prosa* (white) and the Air Force Village (red).

South American coatis were captured in two areas within the city of Campo Grande, located in the state of Mato Grosso do Sul, Brazil, in the central‐western region of the country: (i) *Parque Estadual do Prosa* (PEP), a conservation unit in the core of Campo Grande with 135 ha and (ii) a private area of the Air Force Village (AFV), a complex of 197 ha divided into a military operational zone and a residential area inhabited by at least 730 humans with their domestic animals (Barreto et al., 2024). The predominant vegetation in Campo Grande is Cerrado, a tropical savanna that is characterised by hot, semi‐humid summers and distinct seasonal variations, with a dry period from April to October and a rainy season from November to March (Barreto et al., 2021) (Figure 1).

### Trapping procedures

South American coatis were captured every 3 weeks for 10 consecutive days from March 2018 to April 2019 using 80 metal traps (1 m × 0.40 m × 0.50 m), with 40 traps per area. The traps were strategically placed to ensure human accessibility and shade, covering most of the PEP and AFV areas. Captured animals were anaesthetised with a combination of tiletamine hydrochloride and zolazepam hydrochloride (Telazol, Zoetis® New Jersey, USA) at approximately 6 mg/kg, intramuscularly. After chemical restraint, animals were marked with numbered colour‐coded ear tags, and a microchip was implanted subcutaneously between the shoulder blades for future identification. Each animal was measured, and its age was estimated according to Olifiers et al. (2010). Blood was collected from the femoral vein using tubes containing EDTA (ethylenediamine tetraacetic acid), then transferred to RNAse/DNAse‐free cryotubes and stored at −80°C until molecular analysis. A thorough inspection for ectoparasites was conducted by a single person on the entire body of the individual for 3 min. Ticks were removed with forceps, stored in 100% alcohol and identified to the genus (larvae) or species (nymphs and adults) using a stereomicroscope following taxonomic literature (Dantas‐Torres et al., 2019; Martins et al., 2010). All specimens were deposited in the Acari Collection of Instituto Butantan, São Paulo, Brazil.

All experimental procedures were approved by the Instituto Chico Mendes de Biodiversidade (ICMBio) under SISBIO licence 49662‐8, the Ethics Committee on Animal Use of the School of Agricultural and Veterinary Sciences, UNESP (CEUA FCAV/UNESP 06731/19), the Ethics Committee on Animal Use of Universidade Católica Dom Bosco (CEUA UCDB 001/2018) and the Air Force Cooperation Agreement (N°01/GAP‐CG/2018).

### Parasite sampling

DNA was extracted from 200 μL of blood using the Illustra Blood Mini Kit (GE Healthcare®, Chicago, IL, USA), following the manufacturer's instructions. To assess the quality of the extracted DNA and avoid false‐negative results, the DNA samples were tested using PCR targeting the mammalian *gapdh* gene (Birkenheuer et al., 2003). Ultra‐pure sterile water (Life Technologies®, Carlsbad, CA, USA) was used as a negative control in the PCR assays. PCR screening for *Anaplasma*, *Ehrlichia*, *Hepatozoon*, haemotropic *Mycoplasma* and *Neorickettsia* was conducted by targeting specific genes for each genus, as described by Perles, Barreto, de Macedo, et al. (2023), Perles, Barreto, Santos, et al. (2023), Perles, de Macedo, et al. (2022) and Perles, Herrera, et al. (2022).

### Network structure

To examine the relationships among the detected haemoparasites, we used a modularity network metric (M) (Sano et al., 2024). To assess the significance of network metrics, Monte Carlo procedures were employed, comparing the observed metrics with 1000 random matrices generated from null models. We generated them based on the original weighted matrix using the algorithm proposed by Pinheiro et al. (2019). Significance was determined when the network structure deviated significantly from the null model at *p* < 0.05. Subsequently, to evaluate the relative importance of each node in the network structure, we computed the individual role for each South American coati and each parasite species. This involved determining their network functional role, classifying nodes based on their position and significance within the network. The classifications included (i) ultra‐peripheral vertices (all interactions within their module), (ii) peripheral vertices (most interactions within their module), (iii) non‐hub connector vertices (many interactions to other modules), (iv) non‐hub kinless vertices (interactions evenly distributed among all modules), (v) provincial hubs (most interactions within their module), (vi) connector hubs (many interactions to most other modules) and (vii) kinless hubs (interactions homogeneously distributed among all modules) (Mello et al., 2013; Queiroz et al., 2021). For the network construction, we used the ‘igraph’ package (Csardi & Nepusz, 2006), and the host–parasite interaction incidence matrix was handled with the ‘Bipartite’ package (Dormann, 2011; Dormann et al., 2008).

### Relationship between biotic and abiotic variables in infections

A variety of specific statistical models were assessed to investigate the influence of covariates (explanatory variables) on each parasite species' role within South American coatis. The examined variables included: (i) weight; (ii) age; (iii) sex; (iv) quantities of immature ticks collected from each individual (Perles, Martins, et al., 2022); and (v) collection area. Additionally, a full model (all variables) and null model, devoid of any variables, were established. The species role of each coati was treated as a categorical variable, representing distinct categories. To appropriately model this response variable, the analysis was conducted utilising generalised linear models (GLMs) with the multinomial family, which allowed us to account for the multiple, unordered categories effectively. All candidate models were compared in a model selection approach based on the Akaike information criterion corrected (AIC_c_) for small samples (Akaike, 1974), considering as plausible all models with ΔAIC_c_ ≤ 2, using the package ‘AICcmodavg’ version 2.3–1 (Mazerolle, 2020). All data were analysed using R 4.2.1(R Core Team, 2022).

## RESULTS

We constructed the networks analysis using data from 100 individuals: 45 from PEP and 55 individuals from AFV (Figure 1). Only two animals were negative for all haemoparasites surveyed and were excluded from the network analysis. We recorded an overall occurrence mainly by haemotropic *Mycoplasma* (90.00%), followed by *Hepatozoon* (63.00%), *Ehrlichia* (12.00%), *Anaplasma* (4.00%) and *Neorickettsia* (3.00%), the last only recorded in AFV (3/55) (Table 1). We observed an occurrence of 36% of single infections by haemotropic *Mycoplasma* (29.00%) and *Hepatozoon* (7.00%) (Table 2). Regarding *Ehrlichia* infections, we observed a higher percentage of occurrence in individuals collected at AFV (18.18%) compared to PEP (4.44%). Individuals infected with *Anaplasma* (PEP: 4.44%; AFV:3.64%), *Hepatozoon* (PEP: 66.67%; AFV: 60.00%) and haemotropic *Mycoplasma* (PEP: 93.33%; AFV: 87.27%) showed similar percentages of occurrence between the areas (Table 1). A total of 2242 ticks were collected, including 838 larvae identified as *Amblyomma* spp. Among the nymphs, 1241 were classified as *Amblyomma sculptum* Berlese and 150 as *Amblyomma dubitatum* Neumann. Considering adult ticks, we collected 13 individuals, comprising three males and five females of *A. sculptum*, as well as two males and three females of *Amblyomma ovale* Koch.

**TABLE 1:** Number of *Nasua nasua* (Carnivora: Procyonidae) Linnaeus South American coati parasitised by tick‐borne haemoparasites in a conservation area (*Parque Estadual do Prosa*; *n* = 45) and in the Air Force Village (*n* = 55) in the municipality of Campo Grande, Mato Grosso do Sul, Brazil.

	*Hepatozoon*	*Neorickettsia*	*Ehrlichia*	*Anaplasma*	Haemotropic *mycoplasma*
Overall	63	3	12	4	90
*Parque Estadual do Prosa*	30	0	2	2	42
Air Force Village	33	3	10	2	48

**TABLE 2:** Single and co‐infections of tick‐borne parasites in *Nasua nasua* Linnaeus (Carnivora: Procyonidae) South American coati captured in a conservation area *Parque Estadual do Prosa* (*n* = 45) and in the Air Force Village (*n* = 55) at the municipality of Campo Grande, Mato Grosso do Sul, Brazil.

Co‐infection / infection	Overall	*Parque Estadual do Prosa*	Air Force Village
*Anaplasma* + *Hepatozoon* + haemotropic *Mycoplasma*	2	1	1
*Ehrlichia* + *Hepatozoon* + haemotropic *Mycoplasma*	4	1	3
*Ehrlichia* + haemotropic *Mycoplasma* + *Neorickettsia*	1	0	1
*Hepatozoon* + haemotropic *Mycoplasma* + *Neorickettsia*	1	0	1
*Anaplasma* + haemotropic *Mycoplasma*	2	1	1
*Ehrlichia* + *Hepatozoon*	3	1	2
*Ehrlichia* + haemotropic *Mycoplasma*	4	0	4
*Hepatozoon* + haemotropic *Mycoplasma*	46	25	21
*Mycoplasma* + *Neorickettsia*	1	0	1
*Hepatozoon*	7	2	5
Haemotropic *Mycoplasma*	29	14	15

**FIGURE 2:**
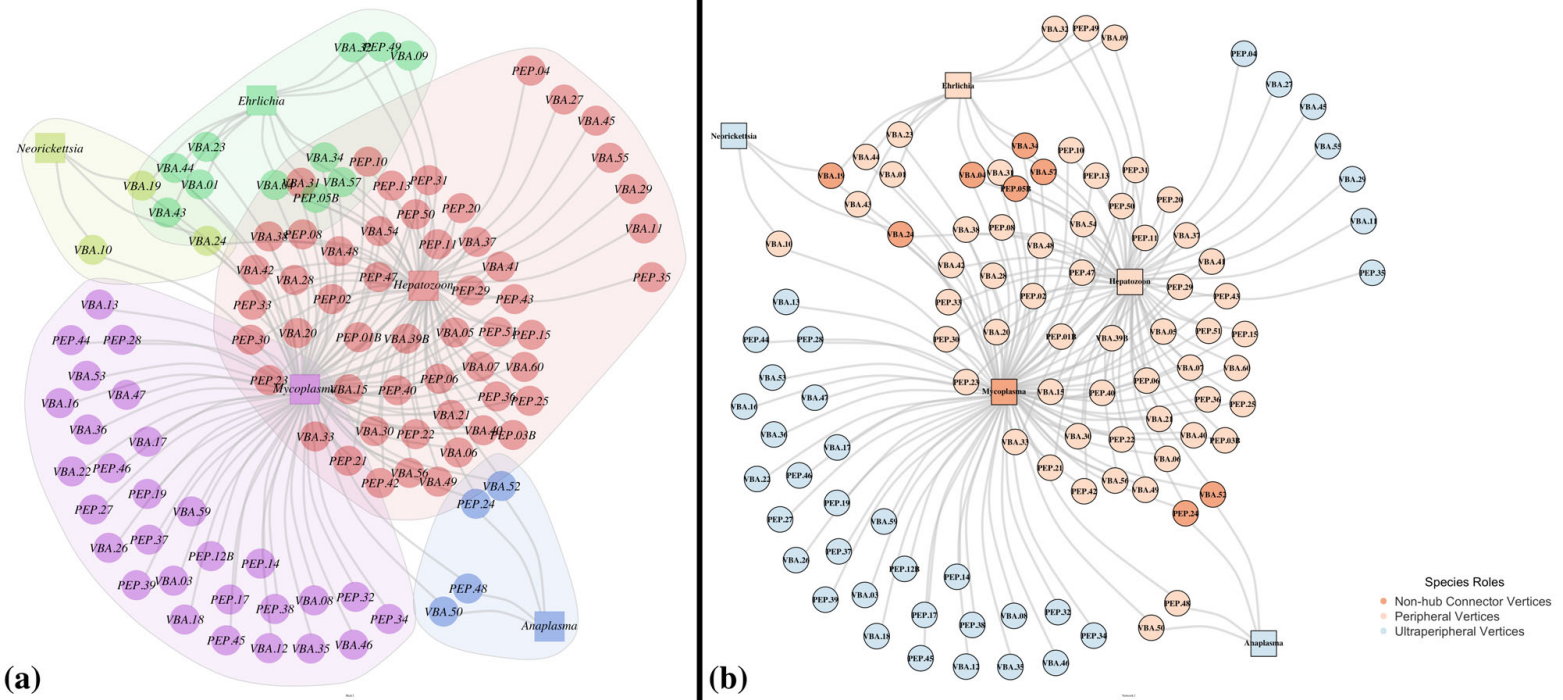
(a) Host–parasite networks of interactions between individual *Nasua nasua* Linnaeus (Carnivora: Procyonidae) South American coati (circle) and parasites (square) using the presence of parasite species in each host individual (disregarding the sampling areas), captured in forest fragments in the municipality of Campo Grande, Mato Grosso do Sul, Brazil. The colour cloud around groups of nodes represents the interaction modules identified using the Beckett modularity detection algorithm, reflecting which South American coati belongs to each parasite species group. The red cloud comprises the *Hepatozoon* group, the blue cloud comprises the *Anaplasma* group, while the veridian green cloud reflect the *Ehrlichia* group, and the leaf‐green cloud is related to *Neorickettsia* group. (b) Host–parasite networks of roles between South American coatis and parasites using the presence of parasite species in each host individual (disregarding the sampling areas), captured in forest fragments in the municipality of Campo Grande, Mato Grosso do Sul, Brazil. The colour of nodes represents species roles.

The network presented low modularity values (Network I: Qw modularity = 0.27, *p* = 0.96, Modules = 05) (Figure 2a). The South American coatis functional role was categorised as 8 non‐hub connector vertices, 56 peripheral vertices and 36 ultra‐peripheral vertices (Figure 2b). Considering the haemoparasites, haemotropic *Mycoplasma* was categorised as non‐hub connector vertices, *Ehrlichia* and *Hepatozoon* as peripheral vertices and *Anaplasma* and *Neorickettsia* as ultra‐peripheral vertices (Figure 2b). None of the biotic variables (weight, age and sex), immature stages of ticks and the sampling locations had any influence on the functional role of *N. nasua* (Table S1).

## DISCUSSION

The network analysis revealed important patterns in the epidemiology and ecology of haemoparasites within a socially structured population of South American coatis. The relatively low modularity observed in the infection network indicates a broad distribution of parasites across the coati population and suggests a diffuse interrelationship between parasites and hosts. Networks with low modularity tend to have fewer distinct subdivisions and higher connectivity among modules, facilitating the horizontal transmission of parasites (Fortuna et al., 2010; Mello et al., 2011). In gregarious animal populations like South American coatis, where social behaviour promotes the transmission of parasites, this wide distribution may be further facilitated, with individuals exhibiting co‐infections. These findings emphasise that social structure, rather than individual characteristics such as age, weight, sex or immature tick loads, is the primary driver of parasite transmission dynamics.

Our analysis showed that neither intrinsic characteristics of the hosts nor the sampled area significantly influenced their functional role within the network. Instead, the social structure of the South American coati population, which is strongly associated with forested areas (Barreto et al., 2024), plays a more significant role in mediating parasite transmission. The presence of non‐hub connector vertices, individuals infected by three different haemoparasites, further supports the idea that the social structure aids in maintaining network cohesion by facilitating interaction among parasites (Fortuna et al., 2010; Mello et al., 2013; Santos & Sano, 2022a). These individuals act as bridges, contributing to the broad distribution of parasites, including consistent infections by *Mycoplasma* and *Hepatozoon*, alongside a variable third parasite (*Ehrlichia*, *Anaplasma* or *Neorickettsia*). The overpopulation of ticks in urban forest fragments of Campo Grande, alongside significant populations of capybaras and coatis, may also play a role in facilitating parasite transmission (Barreto et al., 2021; Serra‐Medeiros et al., 2021). These results suggest that social behaviour within South American coati populations, rather than environmental characteristics or individual traits, drives the transmission dynamics of parasites. Further research is necessary to explore the complex interplay between host social behaviour, individual susceptibility and the distribution of parasites in this system (Godfrey, 2013; Lynsdale et al., 2022; Reisen, 2010).

Considering the haemoparasites evaluated, haemotropic *Mycoplasma* played a central role, acting as a non‐hub connecting vertex in the South American coatis infection network. As haemotropic *Mycoplasma* may be potentially transmitted through saliva (Cannon et al., 2016; Dean et al., 2008; Sidrak et al., 2022), the frequent agnostic interactions observed between South American coatis, combined with potential vector transmission (an alternative route yet to be confirmed [personal communication]), likely result in the high occurrence (90%) detected in the South American coati population at Campo Grande. Considering that *Hepatozoon* is transmitted by ingestion of infected ticks containing sporulated oocysts, our data reinforce the observation recorded by Rucco et al. (2020) that South American coatis eat ticks in an urban park of Campo Grande, Mato Grosso do Sul, Brazil. Although classified as a peripheral vertex, it appeared in 63% of sampled South American coatis. This aligns with epidemiological models that highlight social structure and host behaviour as crucial factors in understanding parasite transmission (Sano et al., 2024; Santos et al., 2021).

*Ehrlichia* was classified as a peripheral vertex, with larger detection in the AFV (18%) rather than in the PSP (4%), likely due to the presence of competent tick vectors (Perles, Martins, et al., 2022; Rikihisa, 2003). Indeed, Perles, Martins, et al. (2022) recorded more immature forms of *Amblyomma* (Ixodida: Ixodidae) in AFV than in PSP. *Anaplasma* and *Neorickettsia* were classified as ultra‐peripheral vertices, as demonstrated by the low molecular occurrences in the South American coatis living in urban parks in Campo Grande, Mato Grosso do Sul. The observed low occurrence of *Anaplasma* in both sampled areas may be related to undetectable bacteraemia recorded in the chronic stages of infection (Lara et al., 2020). On the other hand, *Neorickettsia* was observed only in the AFV, suggesting a localised infection pattern, possibly linked to the presence of paratenic or intermediate host parasitised by digenean organisms (Greiman et al., 2014; Perles, Barreto, de Macedo, et al., 2023).

The interaction of parasites with the South American coati population is influenced by the host's social structure and movement patterns. South American coatis, known for their social groups, enhance intragroup interactions that promote the spread of directly transmitted pathogens like haemotropic *Mycoplasma* (Hirsch, 2011). This behaviour supports network connectivity and is crucial for network cohesion, contributing to the emergence of connector vertices (Godfrey, 2013). The classification of *Hepatozoon*, *Ehrlichia*, *Anaplasma* and *Neorickettsia* as peripheral or ultra‐peripheral reflects their dependence on vectors for transmission. The spread of these parasites is more affected by environmental conditions and vector ecology than by direct host interactions (D'Bastiani et al., 2020). Their fragmented distribution in the South American coati population aligns with ecological theories emphasising the roles of environmental heterogeneity and vector dynamics (Bradley & Altizer, 2007). Haemotropic *Mycoplasma* acts as a non‐hub connector vertex due to its adaptability in maintaining a persistent presence in the population through frequent interactions, based on understanding directly transmitted pathogen epidemiology and ecology within host behaviour (Dobson, 2004).

## CONCLUSION

The presence of non‐hub connector vertices shown by multiple haemoparasites emphasises the interconnected parasite transmission within host populations. This pattern indicates that haemoparasites studied herein may be transmitted through distinct routes, highlighting the direct transmission between individuals with close social interactions and indirect transmission through tick vectors, as well as the ingestion of parasitized paratenic/intermediate hosts. The interaction of haemoparasites with the South American coati population in Campo Grande may be influenced by social and individual behaviours.

## AUTHOR CONTRIBUTIONS

**Filipe Martins Santos:** Conceptualization; data curation; writing – original draft; writing – review and editing; project administration; validation; investigation; methodology; software; formal analysis; supervision. **Nayara Yoshie Sano:** Conceptualization; writing – original draft; writing – review and editing; formal analysis; investigation; methodology. **Livia Perles:** Conceptualization; writing – review and editing; visualization; data curation; validation; methodology. **Marcos Rogério André:** Conceptualization; funding acquisition; writing – review and editing; validation; visualization; resources.

## FUNDING INFORMATION

This work was supported by the following Brazilian research agencies: Coordenação de Aperfeiçoamento de Nível Superior (CAPES — Finance code 001), Conselho Nacional de Desenvolvimento Científico e Tecnológico (CNPq), Financiadora de Estudos e Projetos (FINEP) and FAPESP (Foundation for Research Support of the State of São Paulo) process numbers 2018/02753‐0; 2020/12037‐0). FMS received a fellowship from CAPES (process number 88887.369261/2019–00) and FINEP (process number 01.24.0114.00) NYS received a fellowship from Project Rede Pantanal from the Ministry of Science, Technology, and Innovations of Brazil (FINEP process numbers 01.20.0201.00 and 01.24.0114.00). Productivity grant to MRA (CNPq process number 303701/2021‐8). LP received a scholarship from CNPq and FAPESP (process number 2019/15150‐4).

## CONFLICT OF INTEREST STATEMENT

The authors declare no conflicts of interest.

## Supporting information


**Data S1.** Models used to explore the variables that could influence the functional role in *Nasua nasua* captured in forest fragments in the municipality of Campo Grande/MS. K, number of parameters; AIC_c_, Akaike information criterion; ΔAIC_c_, delta Akaike information criterion; AIC_c_Wt, Akaike Weight; Cum.Wt, cumulative Akaike weight.

## Data Availability

Data used in this manuscript are available from the Dryad Digital Repository https://doi.org/10.5061/dryad.wpzgmsbzk.
