# Supplementary material for: Host–parasite relationship in urban environments: A network analysis of haemoparasite infections in Nasua nasua Linnaeus (South American coati)
Source: Med Vet Entomol. 2025 Mar 24;39(3):456–63. doi: 10.1111/mve.12803 (PMC12323747; doi:10.1111/mve.12803)
Supplement: Supplementary file 1 — Data S1. Models used to explore the variables that could influence the functional role in Nasua nasua captured in forest fragments in the municipality of Campo Grande/MS. K, number of parameters; AICc, Akaike information criterion; ΔAICc, delta Akaike information criterion; AICcWt, Akaike Weight; Cum.Wt, cumulative Akaike weight. [file MVE-39-456-s001.docx]

**Table S1.** Models used to explore the variables that could influence the functional role in *Nasua nasua* captured in forest fragments in the municipality of Campo Grande/MS. K=Number of Parameters; AICc=Akaike Information Criterion; ΔAICc=Delta Akaike Information Criterion; AICcWt=Akaike Weight; Cum.Wt=Cumulative Akaike Weight.

| **Models** | **K** | **AICc** | **ΔAICc** | **AICcWt** | **Cum.Wt** |
| --- | --- | --- | --- | --- | --- |
| Model Null (Species role ~ 1) | 1.00 | 0.00 | 0.28 | 0.28 | 0.00 |
| Model 6 (Species role ~ area) | 2.00 | 0.60 | 0.21 | 0.48 | 0.60 |
| Model 3 (Species role ~ age) | 2.00 | 0.61 | 0.20 | 0.69 | 0.61 |
| Model 2 (Species role ~ weight) | 2.00 | 2.00 | 0.10 | 0.79 | 2.00 |
| Model 4 (Species role ~ sex) | 2.00 | 2.01 | 0.10 | 0.89 | 2.01 |
| Model 5 (Species role ~ tick infestation) | 2.00 | 2.04 | 0.10 | 0.99 | 2.04 |
| Model 7 (Species role ~ all variables) | 6.00 | 6.80 | 0.01 | 1.00 | 6.80 |
